# Individual and community-level factors of abortion in East Africa: a multilevel analysis

**DOI:** 10.1186/s13690-022-00938-8

**Published:** 2022-08-09

**Authors:** Tesfa Sewunet Aalmneh, Adugnaw Zeleke Alem, Gebrekidan Ewnetu Tarekegn, Tilahun Kassew, Bikis Liyew, Bewuketu Terefe

**Affiliations:** 1 Department of Epidemiology and Biostatistics, College of Medicine and Health Sciences, Institute of Public Health, University of Gondar, Gondar, Ethiopia; 2 Departments of Psychiatry, College of Medicine and Health Sciences, University of Gondar, Gondar, Ethiopia; 3 School of Nursing, College of Medicine and Health Sciences, Department of Emergency and Critical Care Nursing, University of Gondar, Gondar, Ethiopia; 4 School of Nursing, College of Medicine and Health Sciences, Community Health Nursing Department, University of Gondar, Gondar, Ethiopia

**Keywords:** Abortion, Reproductive age women, East Africa, Demographic and Health Survey, Maternal mortality, Multilevel analysis

## Abstract

**Background:**

Abortion is one of the top five causes of maternal mortality in low and middle-income countries. It is associated with a complication related to pregnancy and childbirth. Despite this, there was limited evidence on the prevalence and associated factors of abortion in East African countries. Therefore, this study aimed to investigate the prevalence and associated factors of abortion among reproductive-aged women in East African countries.

**Methods:**

The Demographic and Health Surveys (DHS) data of 12 East African countries was used. A total weighted sample of 431,518 reproductive-age women was included in the analysis. Due to the hierarchical nature of the DHS data, a multilevel binary logistic regression model was applied. Both crude and Adjusted Odds Ratio (AOR) with 95% Confidence Interval (CI) was calculated for potential associated factors of abortion in East Africa. In the final model, variables with a *p* value < 0.05 were declared as statistically significant factors of abortion.

**Results:**

Around 5.96% (95%CI: 4.69, 7.22) of reproductive-aged women in East Africa had a history of abortion. Alcohol use, tobacco or cigarette smoking, being single, poorer wealth index, currently working, traditional family planning methods, and media exposure were associated with a higher risk of abortion. However, higher parity, having optimum birth intervals, and modern contraceptive uses were associated with lower odds of abortion.

**Conclusions:**

The prevalence of abortion among reproductive-aged women in East Africa was high. Abortion was affected by various socio-economic and obstetrical factors. Therefore, it is better to consider the high-risk groups during the intervention to prevent the burdens associated with abortion.

## Background

Globally, many women die due to pregnancy and birth-related complications; nearly 99.0% of maternal death occurs in low and middle-income countries [1]. Among the top five causes, abortion is one of the fundamental causes of maternal mortality in low and middle-income countries [2]. Abortion may occur spontaneously or intentionally; the latter is also called induced abortion, which may be safe or unsafe. Abortion (incredibly unsafe) may have serious health consequences and cause complications such as hemorrhage, sepsis, and uterine perforation [3, 4].

Globally, around 210 million women become pregnant each year. Of these, 80 million pregnancies are unwanted. Forty-six million are terminated from these pregnancies, and 19 million ends with unsafe abortion [4–6]. Maternal death associated with abortion, especially the unsafe, accounts for 13% of maternal death globally, that was 37 deaths per 100,000 live births in Sub-Sharan Africa (SSA) and 12 per 100,000 in South Asia [7].

The finding of previous studies across the world revealed that several factors are associated with abortion. The risk for abortion is higher for teenagers and women older than 35 years old [8, 9], not attending formal education [10, 11], residents [12, 13], poor individuals [14], single women [15], women who engage in physical activity [16, 17], pervious delivery by cesarean Sect. [18], short interpregnancy interval [19, 20], low parity [9], maternal under-nutrition [21, 22], use of substance like cigarette and tobacco [23–25], and multiple pregnancies [26–28].

This is one of the benefits of including multinational abortion data in this analysis. It serves as a guide to the plans and interventions of various international, continental and national organizations to enable them which region is severely affected and needs urgent further research and policy amendments [29, 30]. Despite abortion being associated with pregnancy and birth-related complications and the vital cause of maternal mortality, especially in low- and middle-income countries, to the best of our knowledge, there is no pooled data that determine the prevalence and associated factors of abortion in East African countries that is the region where low- and middle-income countries founded.

Therefore, this study aimed to investigate the prevalence and associated factors of abortion among reproductive-aged women in East African countries. Conducting this study will help decide maternal health based on the best available scientific evidence.

## Methods

### Data source and sampling procedure

We used the most recent Demographic and Health Survey (DHS) data of 12 East African countries conducted from 2008 to 2018 to determine the magnitudes and associated factors of abortion in East Africa.

The DHS surveys are routinely collected every five years across low-and middle-income countries using structured, pretested, and validated questionnaires. The DHS surveys follow the same standard procedure sampling, questionnaires, data collection, and coding, making multi-country analysis possible. The DHS survey employs a stratified two-stage cluster sampling technique. In the first stage, clusters/enumeration areas (EAs) were randomly selected from the sampling frame (i.e., they are usually developed from the available latest national census). In the second stage, systematic sampling was employed on households listed in each cluster or EA. Interviews were conducted in selected households with target populations (women aged 15–49 and men aged 15–64). All reproductive-aged women who gave birth in the five years preceding the most recent DHS of 12-east African countries were included in this study. However, a woman with missing data on the outcome variable (abortion) was excluded from the study. This includes women are infertile, sexually inactive and did not have pregnancy history. Any missing data at any outcome variable was treated by applying various missing data management techniques according to the instruction of the guide to DHS statistics [31]. A total weighted sample of 431,518 reproductive-age women was included (Table 1).

**Table 1:** Countries, sample size, and survey year of Demographic and Health Surveys included in the analysis for 12 East African countries

Country	Survey year	Sample size
Burundi	2016/17	41,129
Ethiopia	2016	41,526
Kenya	2014	33,705
Comoros	2012	10,344
Madagascar	2008/09	45,735
Malawi	2015/16	61,613
Mozambique	2011	38,141
Rwanda	2014/15	26,202
Tanzania	2015/16	31,198
Uganda	2016	47,913
Zambia	2018	34,536
Zimbabwe	2015	19,482

### Variables of study

The outcome variable for this study was abortion among the reproductive-aged, which was derived from the DHS question, "have you ever had a terminated pregnancy.” It was dichotomized as “Yes” if a woman had experienced abortion, either spontaneous or induced (termination of pregnancy before seven completed months of pregnancy), and “No” if a woman hadn't experienced abortion.

The independent variables of the study includes community level variables such as residence (urban and rural) and distance to health facility ( not big problem and a big problem), and individual level variables like maternal age (less than 20, 20–34 and greater or equal to 35), education status (no formal education, primary, secondary and higher), marital status (single, married, divorced, widowed and separated), wealth index (poorest, poorer, middle, richer and richest) which was calculated by principal component analysis for urban and rural areas separately based on their asset, currently working (yes and no), mass media (reproductive aged women were considered as exposed to mass media when they watch either television or radio at least once per wee k otherwise considered as not exposed), smoking (yes and no), preceding birth interval (less than 24 months/not optimum and greater or equal to 24 months/optimum), alcohol use (yes and no), contraceptive use (non- user, modern and traditional (when the participant uses either abstinence from intercourse, withdrawal method or calendar method)) and parity (less than 5 births and greater than or equal to 5 births).

### Data management and statistical analysis

The variables of the study were extracted, cleaned, and recoded using STATA version 14. The data were weighted using sampling weight during any statistical analysis to adjust for unequal probability of selection due to the sampling design used in DHS data. Hence, the representativeness of the survey results was ensured.

A two-level multivariable binary logistic regression analysis was used to estimate the effect of explanatory variables on abortion. The data has two levels with a group of J EAs and within-group j (j = 1, 2…, J), a random sample nj of level-one units (reproductive-aged woman). The response variable is denoted by;

- Y_ij_ = 0 if the i^th^ mother was in the j^th^ EA’s had a history of abortion
- 1 if i^th^ mother was in the j^th^ EAs had no history of abortion

So, appropriate inferences and conclusions from this data require proper modeling techniques like multilevel modeling, which contain variables measured at different levels of the hierarchy, to account for the nested effect [32]. Four models were fitted for the data. The first model was an empty model without any explanatory variables to calculate the extent of cluster variation in abortion. Variations between clusters (EAs) were assessed by computing Intra-class Correlation Coefficient (ICC), Proportional Change in Variance (PCV), and Median Odds Ratio (MOR). The ICC is the proportion of variance explained by the grouping structure in the population. Whereas PCV measures the total variation attributed to individual and community level factors in the multilevel model as compared to the null model [33]. The MOR is also defined as the median value of the odds ratio between the cluster at high risk and the cluster at lower risk of abortion when randomly picking out two clusters (EAs). The second model was adjusted with community-level variables only; the third model was adjusted for individual-level variables only, while the fourth was fitted with both individual and community-level variables. These four models were compared by using deviance (-2LLR), and the model with the lowest deviance was selected as the best-fitted model for the data.

Variables with a *p*-value ≤ 0.2 in the bi-variable analysis were considered for the multivariable analysis. In the multivariable multilevel binary logistic model, the Adjusted Odds Ratio (AOR) with 95% Confidence Interval (CI) of the best-fitted model was reported to identify the associated factors of abortion. The statistical significance for the final model was set at *p* < 0.05.

### Ethical consideration

This study is a secondary data analysis from the DHS data of 12 East African countries (Burundi, Ethiopia, Kenya, Comoros, Madagascar, Malawi, Mozambique, Rwanda, Tanzania, Uganda, Zambia, and Zimbabwe), so it does not require ethical approval. For conducting this study, online registration and request for measure DHS were conducted. The dataset was downloaded from DHS online archive (http://www.dhsprogram.com) after getting approval to access the data.

## Results

### Background characteristics

A total of 431,518 reproductive-aged women were included in the study. Of those, 30,285 (7.02%) women had a history of terminated pregnancy. The majority of the participants, 345,941 (80.17%), were rural dwellers. Less than one-third of participants, 131,830 (30.55%), were not educated. The majority of the participants, 357,323 (82.81%), were married.

Regarding the behaviors of the participants, 238,453 (55.26%) had no exposure to mass media, 18,696 (4.33%) participants were smoking cigarettes or tobacco, 70,774 (16.40%) participants were using alcohol, and 259,159 (60.06%) were not using any contraceptive method (Table 2).

**Table 2:** Background characteristics of the study participants in East Africa

Variables	Weighted frequency	Percentage (%)
**Residence**		
Urban	85,577	19.83
Rural	345,941	80.17
**Distance to the health facility**		
Not a big problem	225,089	55.14
A big problem	183,147	44.86
**Age (years)**		
< 20	8,010	1.86
20–35	217,131	50.32
> 35	206,377	47.83
**Education status**		
No	131,830	30.55
Primary	224,915	52.12
Secondary	65,446	15.17
Higher	9,329	2.16
**Marital status**		
Single	9,961	2.31
Married	357,323	82.81
Divorced	21,809	5.05
Widowed	16,328	3.78
Separated	26,098	6.05
**Wealth index**		
Poorest	96,539	22.37
Poorer	90,637	21.00
Middle	88,611	20.54
Richer	84,887	19.67
Richest	70,841	16.42
**Currently working**		
No	132,892	30.80
Yes	298,590	69.20
**Mass media exposure**		
No	238,453	55.26
Yes	193,065	44.74
**Smoking**		
No	412,820	95.67
Yes	18,696	4.33
**Preceding birth interval (months)**		
< 24	82,713	26.52
≥ 24	229,216	73.48
**Alcohol use**		
No	360,744	83.60
Yes	70,774	16.40
**Contraceptive use**		
Non- user	259,159	60.06
Traditional	14,932	3.46
Modern	157,428	36.48
**History of terminated pregnancy**		
No	401,233	92.98
Yes	30,285	7.02
**Parity**		
1–4	188,352	43.65
5 +	243,167	56.35

### The prevalence of abortion in East Africa

**Fig. 1:**
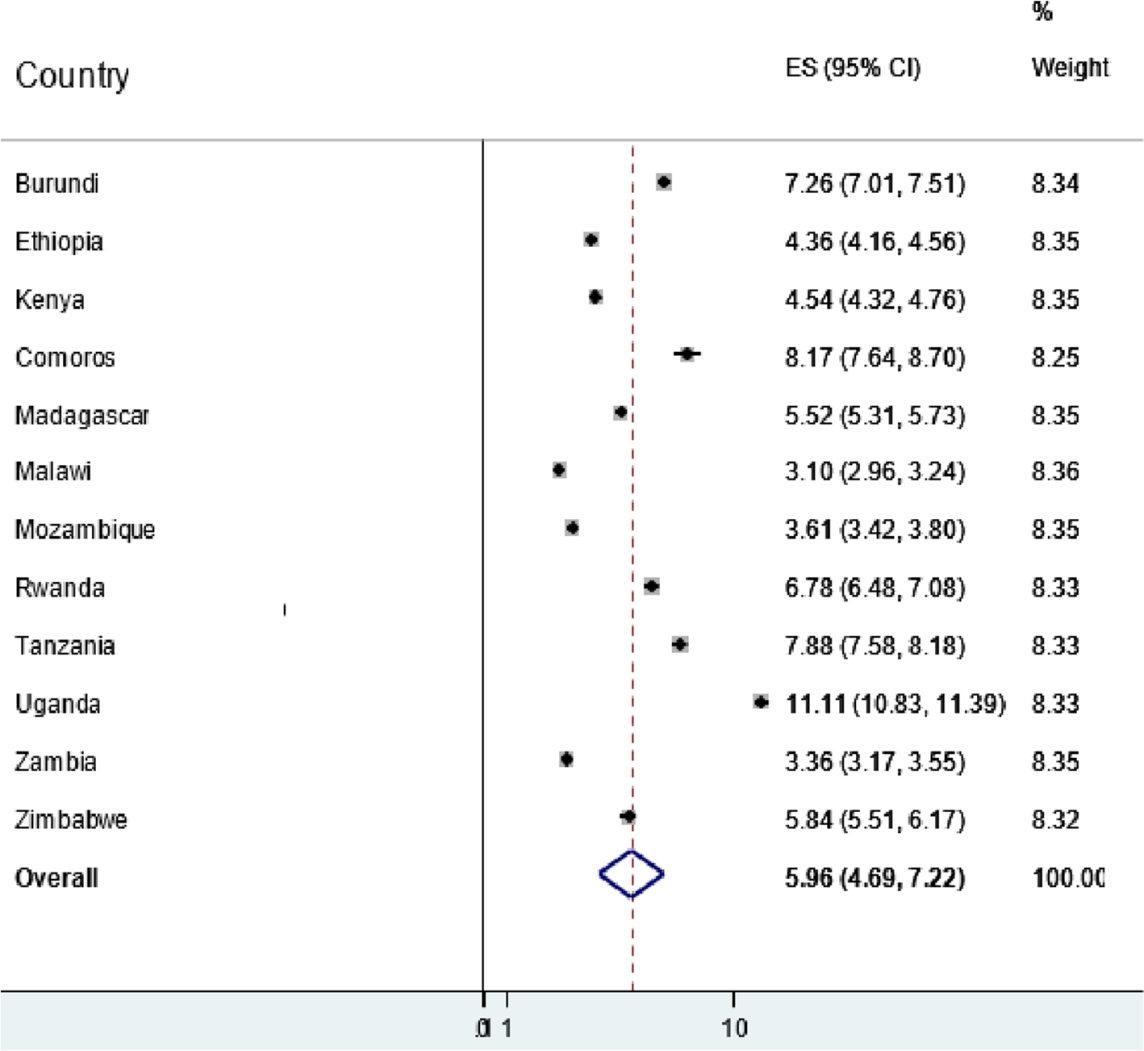
The prevalence of abortion in East African Countries

In East African countries, the pooled prevalence of abortion among reproductive-aged women was 5.96% (95%CI: 4.69, 7.22). The prevalence of abortion in East African countries ranges from 3.10% (95%CI: 2.96, 3.24) in Malawi to 11.11% (95%CI: 10.83, 11.39) in Uganda (Fig. 1).

### Random effect analysis and model comparison

In the first model (empty model), the ICC indicated that about 17.11% of the total variability for abortion was due to differences between clusters/EA, with the remaining unexplained 82.99% attributable to the individual differences. In addition, the median odds ratio also revealed that abortion was heterogeneous among clusters. It was 2.19 in the first model, which implies the women within the cluster having a higher risk for abortion had a 2.19 times higher chance of having an abortion as compared with children within a cluster having a lower risk if women were selected randomly from two different clusters (EAs). Regarding PCV, about 39.70% of the variability in abortion was explained by the full model. Besides, Model IV was selected as the best-fitted model (which had the lowest deviance) (Table 3).

**Table 3:** Multilevel analysis of factors associated with abortion among reproductive-aged women in East Africa

**Variables**	Model 1	Model 2	model 3	Model 4
	AOR (95%CI)	AOR (95% CI)	AOR (95% CI)	AOR (95% CI)
**Residence**				
Urban		1		1
Rural		0.89(0.87,0.93)		0.98(0.93,1.03)
**Distance to the health facility**				
Not a big problem		1		1
A big problem		0.96(0.93, 0.98)		0.98(0.96,1.02)
**Marital status**				
Single			1	1
Married			0.44(0.36,0.54)	0.45(0.37,0.54)*
Divorced			0.40(0.37,0.44)	0.41(0.37,0.44)*
Widowed			0.56(0.51,0 .62)	0.54(0.48,0.59)*
Separated			0.81(0.75, 0.86)	.84(0.78,0.89)*
**Wealth index**				
Poorest			1	1
Poorer			0.92(0.87, 0.96)	0.93(0.88,0.97)*
Middle			1.01(0.96, 1.05)	0.99(0.95,1.05)
Richer			0.99(0.95, 1.04)	0.98(0.93,1.03)
Richest			0.97(0.92,1.03)	0.96(0.89,1.07)
**Currently working**				
No			1	1
Yes			1.39(1.35, 1.45)	1.43(1.38,1.49)*
**Mass media exposure**				
No			1	1
Yes			1.25(1.21, 1.29)	1.26(1.21,1.30)*
**Smoking**				
No			1	1
Yes			1.25(1.17, 1.34)	1.24(1.16,1.32)*
**Preceding birth interval (months)**				
< 24			1	1
≥ 24			0.97(0.94, 1.01)	0.96(0.93,0.99)*
**Alcohol use**				
No			1	1
Yes			1.15(1.10. 1.19)	1.13(1.08,1.18)*
**Contraceptive use**				
Non- user			1	1
Traditional			1.14(1.06, 1.22)	1.17(1.08,1.26)*
Modern			0.63(0.61,0 .65)	0.63(0.61,0.65)*
**Parity**				
1–4			1	1
5 +			0.92(0.89,0.95)	0.92(0.89,0.95)*
Community level variance	0.68(0.59, 0.78)	0.67(0.58,0.76)	0.46 (0.41,0. 54)	0.41(0.38,0.52)
ICC (%)	17.11	16.74	12.27	11.08
MOR	2.19	2.18	2.01	1.97
PCV (%)	reference	1.47	32.35	39.70
Deviance	189,150	179,262	133,090	126,090

*AOR* Adjusted Odds Ratio, *CI* Confidence Interval, *ICC* Intra-class Correlation Coefficient, *MOR* Median Odds Ratio, *PCV* Proportional Change in Variance

^*^p-value < 0.05

### Factors associated with abortion

In the bi-variable multilevel analysis, all of the explanatory variables (both individual level and community level variables) except maternal age and educational status showed a statistically significant association with abortion at a *p*-value of < 0.20.

In the final model, marital status, wealth index, current working status, substance use (either cigarettes or tobacco), alcohol use, contraceptive use, mass media exposure, parity, preceding birth interval, and parity were significantly associated with abortion (*p* ≤ 0.05).

The likelihood of having a history of abortion was 7% (AOR = 0.93; 95%CI: 1.11, 1.30) lower for women with poorer wealth index as compared to women with poorest wealth index quantile. As compared to single reproductive-aged women, the odds of having abortion history were 55% (AOR = 0.45, 95%CI: 0.37,0.54), 59% (AOR = 0.41, 95%CI: 0.37,0.44), and 46% (AOR = 0.54, 95%CI: 0.48,0.59),16% (AOR = 0.84, 95%CI: 0.78,0.89) lower for married, divorced, widowed and separated reproductive-aged women respectively. Regarding currently working status, the odds of having an abortion among women that were currently working was 1.43 (AOR = 1.43, 95%CI: 1.38,1.49) times their counterparts. Moreover, the chance of having an abortion history was 4% (AOR = 0.96, 95%CI: 0.93,0.99) and 8% (AOR = 0.92, 95%CI:0.89,0.95) lower chance for women having birth space greater than or equal to 24 months and giving birth to five or more children, respectively as compared to their counterparts.

This study also revealed that abortion was significantly associated with sociodemographic and other factors, which implies the odds of having an abortion were 1.24 (AOR = 1.24, 95%CI:1.16,1.32) times for substance users as compared to non-users, 1.13 times (AOR = 1.13, 95%CI:1.08,1.18) for alcohol users as compared to non-users, 1.17 (AOR = 1.17, 95%CI:1.08,1.26) times for traditional contraceptive users and 33% (AOR = 0.63,95%CI: 0.61,0.65) lower chance for modern contraceptive users as compared to non-users. Regarding mass media exposure, the chance of experiencing abortion was 26% (AOR = 1.26:95%CI, 1.21,1.30)) lower for reproductive-age women who were exposed to mass media as compared to those who were not exposed (Table 3).

## Discussion

This study aimed to assess the prevalence and associated factors of abortion in East African countries using recent DHS data of East African countries. According to the findings of this study, abortion was found to be a significant public health problem in East Africa. The higher prevalence of abortion in this study is in line with studies conducted in Ghana, Mozambique, and Ethiopia [13, 34–36]. The higher burden of abortion in East Africa could be explained by inadequate coverage and access to family planning services, higher magnitudes of unwanted pregnancy, and higher burdens of acute and chronic malnutrition among reproductive-aged women [37–39].

This study showed that media exposure was a significant predictor associated with an increased chance of abortion. The finding of this study agrees with other studies in Ethiopia, Ghana, and Mozambique [34, 35]. The possible explanation for the discovery could be a woman who has exposed to media is might have a piece of information about how and where to terminate a pregnancy. In addition, these women might be aware of available laws related to abortion and less likely to be stigmatized by society [40].

This study revealed that the risk of abortion was higher among women who drink alcohol and substance users as compared to their counterparts. The finding of this study was supported by studies done in different parts of the world [24, 25, 41–43]. This finding could be related to alcohol consumption, and substance use is a potential risk for congenital anomalies [44, 45]. A fetus with congenital anomalies had a high risk of adverse pregnancy outcomes, including abortion [46]. Moreover, substance users such as tobacco or cigarette and those who drink alcohol have experienced unwanted/unplanned pregnancies that usually end up with abortion [47, 48].

This study also shows that contraceptive use was significantly associated with a history of abortion among reproductive-aged women. The odds of having an abortion were higher among traditional contraceptive users and lower for modern contraceptive users as compared to women who were not using any type of contraception/family planning method. The finding of this study was supported by previous studies done in sub-Saharan African countries [49]. The possible justification for this finding could be the risk of unwanted/unplanned pregnancy, which is usually terminated before the fetus reaches the age of viability, was higher among traditional users and lower for modern contraceptive users [50, 51].

The likelihood of having an abortion history was lower for mothers having a higher (five or more) number of children and mothers giving birth with an interval of 24 or more months as compared to their reference groups. This finding was supported by previous studies [10, 52]. This could be explained by mothers with high parity may better knowledge regarding menstrual cycles and utilization of maternal health services such as family planning. These mothers may also know that contraceptive use is the best measure to limit the number of children and increase birth space.

The main strength of this study was the use of weighted, representative large datasets of East African countries with an advanced statistical analysis technique that accounts for the correlated nature of DHS data, which enables us more precise estimates and standard errors. Moreover, the result of this study could support policymakers, clinicians, and programmers in designing interventions for preventing abortion in the region. However, this study is not free from limitations. Since the DHSs are cross-sectional surveys, we cannot establish a cause-and-effect relationship between the different independent variables and abortion. Moreover, since the data were collected through interviews, there might be a possibility of recall bias. This study also analyzes without separating spontaneous and induced abortion. Due to the varying timeliness of the data available, this study evaluated and contrasted nations that are not comparable without considering time variant as an independent variable. This may affect the findings of this study. So, attention should be given while using the conclusions of this study.

## Conclusion

Despite the fact that maternal mortality has decreased over the past few years in East Africa, abortion which is the primary cause of maternal mortality is still a significant public health problem. Substance use such as alcohol, tobacco, or cigarette smoking, being single, currently working, use of traditional family planning method, and media exposure was positively associated factors of abortion. However, higher parity, having optimum birth interval, and modern contraceptive use was protective factor of abortion. Therefore, it is better to consider the high-risk groups to prevent abortion among reproductive-aged women during the intervention.

## Data Availability

All relevant data are within the manuscript and its Supporting Information files.

## Abbreviations

AOR: Adjusted Odds Ratio
AOR: Adjusted Odds Ratio
ICC: Intra-class Correlation Coefficient
CI: Confidence Interval
DHS: Demographic and Health Survey
MOR: Median Odds Ratio
MM: Mass Media exposure
WHO: World Health Organization
